# Online Medical Education During the COVID-19 Pandemic: Experience and Perception of Second-Year Bachelor of Medicine and Bachelor of Surgery (MBBS) Students in a Tertiary Care Hospital in Assam, India

**DOI:** 10.7759/cureus.65399

**Published:** 2024-07-25

**Authors:** Pranab Das, Dolly Roy, Nivedita Saha

**Affiliations:** 1 Department of Pharmacology, Silchar Medical College and Hospital, Silchar, IND

**Keywords:** e-learning, covid-19 pandemic, medical education, information technology, online learning

## Abstract

Introduction

The onset of the COVID-19 pandemic-imposed changes in educational practices worldwide. It forced medical institutions to adapt to an online teaching mode, which has advantages and disadvantages. The present study aims to investigate the perceptions of medical students regarding online learning during COVID-19.

Purpose

This study assesses whether online teaching methods are feasible, suitable, preferred, and effective compared to traditional in-class teaching for Bachelor of Medicine and Bachelor of Surgery (MBBS) students.

Methodology

It is a cross-sectional descriptive study conducted on 120 second-year MBBS students of Silchar Medical College, Silchar, India. Using Google Forms, a closed-ended pre-formed questionnaire was distributed to the students to get feedback on the advantages and challenges of online learning. The respondents were prompted to respond to the questions on a Likert scale ranging from 1 to 5. The data were analyzed using Excel 16 (Microsoft® Corp., Redmond, WA).

Results

The majority of the participants positively supported the feasibility, suitability, utility, and effectiveness of online learning. The most preferred online web conferencing platform for e-learning in the survey was Google Classroom. Despite the advantages, online medical education was limited by network-related issues and a lack of socializing with peers. Moreover, looking forward, about 90% of the students preferred online or a combination of online and classroom teaching.

Conclusion

The study highlights the positive attitude toward online education among second-year MBBS Students in a tertiary care hospital in Assam. It provides valuable insights into the challenges faced in e-learning in medical education, forming the groundwork for devising future education strategies.

## Introduction

The COVID-19 pandemic has created a paradigm change in medical education concepts worldwide. Medical schools have been forced to close abruptly because of the COVID-19 pandemic, temporarily stopping classroom teaching. Students were likely to be psychologically distressed and affected by this situation [1]. Online learning, characterized by integrating electronic resources, particularly computer technology and internet-based platforms, emerged as a prominent solution amidst this crisis. This transition from traditional face-to-face instructions to virtual learning environments became imperative to minimize the risk of viral transmission inherent in physical gatherings within educational settings. Many institutions have changed to alternative teaching methods that utilize digital media [2]. Despite the compelling imperative of online learning in maintaining social distancing measures and attenuating the spread of infectious diseases, its implementation is not without challenges. Several studies suggest that students may encounter impediments in fully harnessing the educational benefits of this mode of instruction. Nevertheless, notwithstanding these obstacles, online learning remains a pragmatic and indispensable strategy for ensuring the continuity of educational provision while prioritizing public health imperatives amid extraordinary circumstances such as those precipitated by the COVID-19 pandemic. Traditionally, medical education and clinical training rely on classroom teaching and practical training; however, post-COVID, many educational institutions have accepted online education as a viable method of education [3].

Many colleges globally promote e-learning as the future mode of teaching [4]. However, recent research implies that a fundamental restriction of online learning is the absence of teacher-student interaction and network-related issues [5,6]. This was particularly difficult in a country like India with limited resources due to a lack of expensive technology, high-speed network connections, and a shortage of teachers with the necessary skills for online classes. It could be an overstatement to signify that COVID-19 has been a lifetime instance for medical instructors and college students [3].

Even though online learning is becoming more popular around the world, Indian medical education had never used it before on a large scale before the pandemic struck [7]. Several studies have examined the impact of the COVID-19 pandemic on educational methodologies. However, only limited studies focus specifically on medical students in India. The study aims to contribute to the existing pool of information by assessing the advantages and disadvantages of online teaching and working on the issues faced by students in India, where infrastructure is limited.

The study's objective is to investigate the experiences and perceptions of second-year medical students regarding online learning during the COVID-19 pandemic and analyze the problems they faced in adapting to online learning.

## Materials and methods

This cross-sectional descriptive study was conducted for one month, from December 2023 to January 2024, in the Silchar Medical College and Hospital (SMC/18.843, Dated: 23/11/2023). A total of 120 second-year MBBS students were enrolled in the study by convenience sampling. The purpose of the study was explained, and written informed consent was procured from all the study participants. The subjects' privacy, anonymity, and confidentiality were maintained.

Inclusion criteria

The study included students who had attended online classes during the COVID-19 pandemic and were willing to participate in the study.

Exclusion criteria

The study excluded students who did not attend online classes throughout the COVID-19 pandemic and/or gave incomplete or invalid answers to the questionnaire.

Data collection

Data were collected using a closed-ended, pre-designed, pre-tested questionnaire adapted from Singh et al. utilized in a survey of e-learning methods in medical education during the COVID-19 pandemic in India using Google Forms [8], and the students were asked to select a single applicable response. The questionnaire was subdivided into three sections. The questionnaire was used to assess the feasibility, suitability, utility, and effectiveness of e-learning, and responses were collected using a 5-point Likert scale. Additionally, data related to the problems faced by the students during online teaching were collected. Lastly, the students were asked to state their preferred web conferencing platforms and perceptions of online teaching in the future.

Statistical analysis

Descriptive statistics was used to analyze the data using Microsoft Excel 2016 (Microsoft® Corp., Redmond, WA).

## Results

A total of 120 second-year Bachelor of Medicine and Bachelor of Surgery (MBBS) students participated in the study, all of whom submitted fully completed questionnaires. Among the participants, 55% were male and 45% were female. Respondents to Likert scales 1 and 2 were referred to as the dissatisfied group, Likert scales 4 and 5 as the satisfied group, and Likert acales 3 as neutral (Table 1).

**Table 1:** Experiences and perceptions of students with online learning (Likert scales 1-5) (n = 120).

Sl. No.	Questions	Dissatisfied Group		Neutral/Equivocal (3), n (%)	Satisfied Group	
		Strongly disagree (1), n (%)	Disagree (2), n (%)		Agree (4), n (%)	Strongly Agree (5), n (%)
Feasibility of online learning						
1	Easily access the internet as needed for online studies.	8 (6.7%)	26 (21.7%)	12 (10%)	57 (47.5%)	17 (14.1%)
Suitability of online learning						
2	More comfortable with online learning at home.	7 (5.8%)	26 (21.7%)	13 (10.8%)	60 (50.0%)	14 (11.7%)
3	More comfortable with offline lectures in class.	15 (12.5%)	59 (49.2%)	13 (10.8%)	23 (19.2%)	10 (8.3%)
4	Learning is similar to online or conventional lectures in lecture halls.	16 (13.3%)	59 (49.2%)	11 (9.2%)	27 (22.5%)	7 (5.8%)
Utility of online learning						
5	Students can easily ask questions if there are problems in understanding an online lecture.	7 (5.8%)	28 (23.3%)	11 (9.2%)	68 (56.7%)	6 (5%)
6	More questions can be asked by the students in online learning.	5 (4.2%)	25 (20.8%)	16 (13.3%)	62 (51.7%)	12 (10%)
7	Satisfied with the organization and preparedness of the class.	8 (6.7)	26 (21.6%)	13 (10.8%)	60 (50%)	13 (10.8%)
8	Students get quick responses from teachers to clear their queries via online mode.	8 (6.6%)	26 (21.6%)	14 (11.7%)	63 (52.5%)	9 (7.5%)
Effectiveness of online learning						
9	Online education would make it easy for slow learners and shy students to interact better.	7 (5.8%)	27 (22.5%)	11 (9.2%)	62 (51.7%)	13 (10.8%)
10	A sufficient number of questions, or Multiple-Choice Questions (MCQs), are asked by teachers to make online learning sessions interactive.	7 (5.8%)	26 (21.7%)	15 (12.5%)	59 (49.2%)	13 (10.8%)
11	It simply takes more time to effectively accomplish tasks in an online environment than in a face-to-face class.	8 (6.7%)	29 (24.2%)	13 (10.8%)	60 (50%)	10 (8.3%)

**Figure 1:**
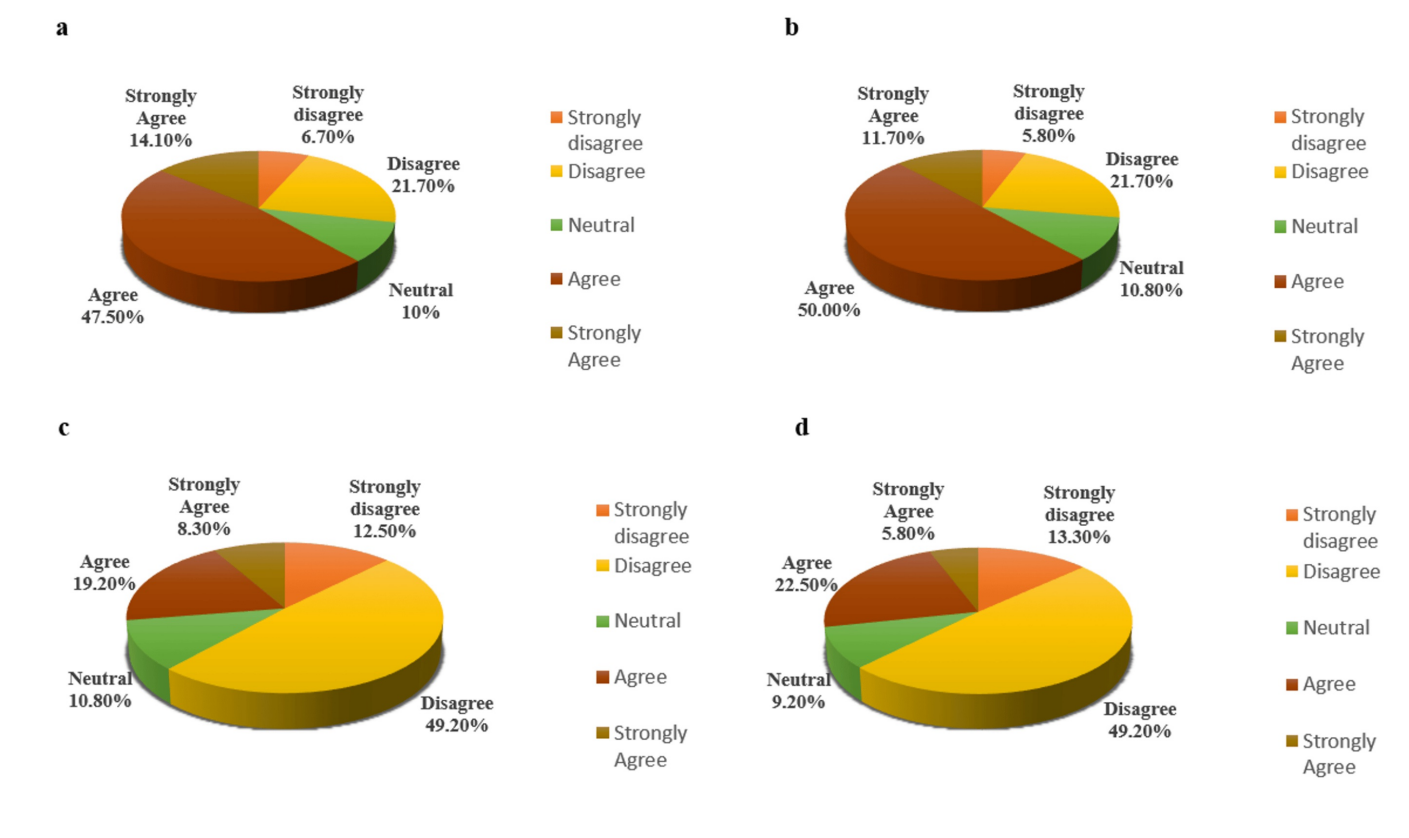
Students’ responses to the questionnaire’s representative parameters associated with online learning: part 1. (a) Easily access the internet as needed for online studies. (b) More comfortable with online learning at home. (c) More comfortable with offline lectures in class. (d) Learning is similar to online or conventional lectures in lecture halls.

**Figure 2:**
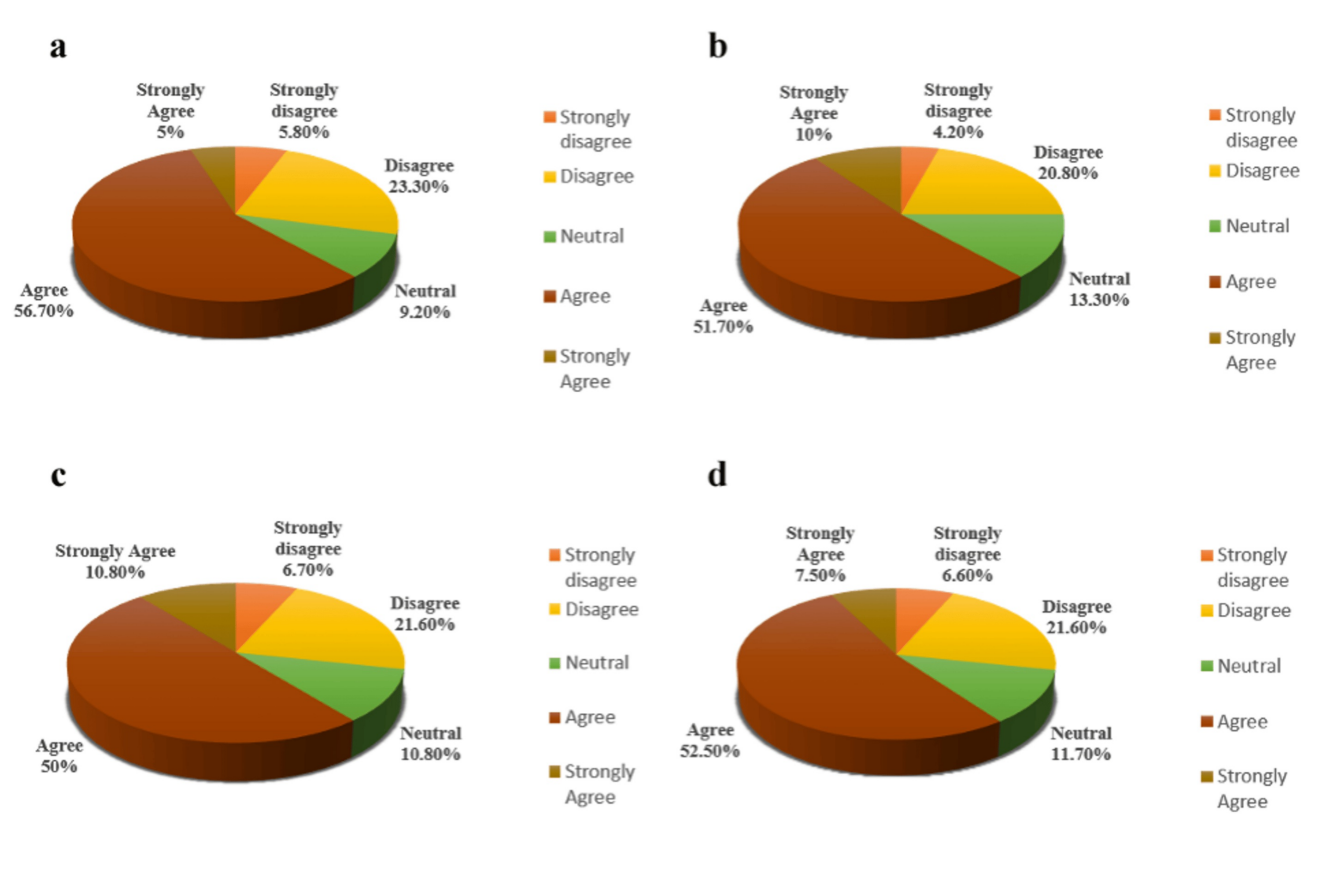
Students’ responses to the questionnaire’s representative parameters associated with online learning: part 2. (a) Students can easily ask questions if they have problems understanding an online lecture. (b) More questions can be asked by the students in online learning. (c) Satisfied with the organization and preparedness of the class. (d) Students get quick responses from teachers to clear their queries via online mode.

**Figure 3:**
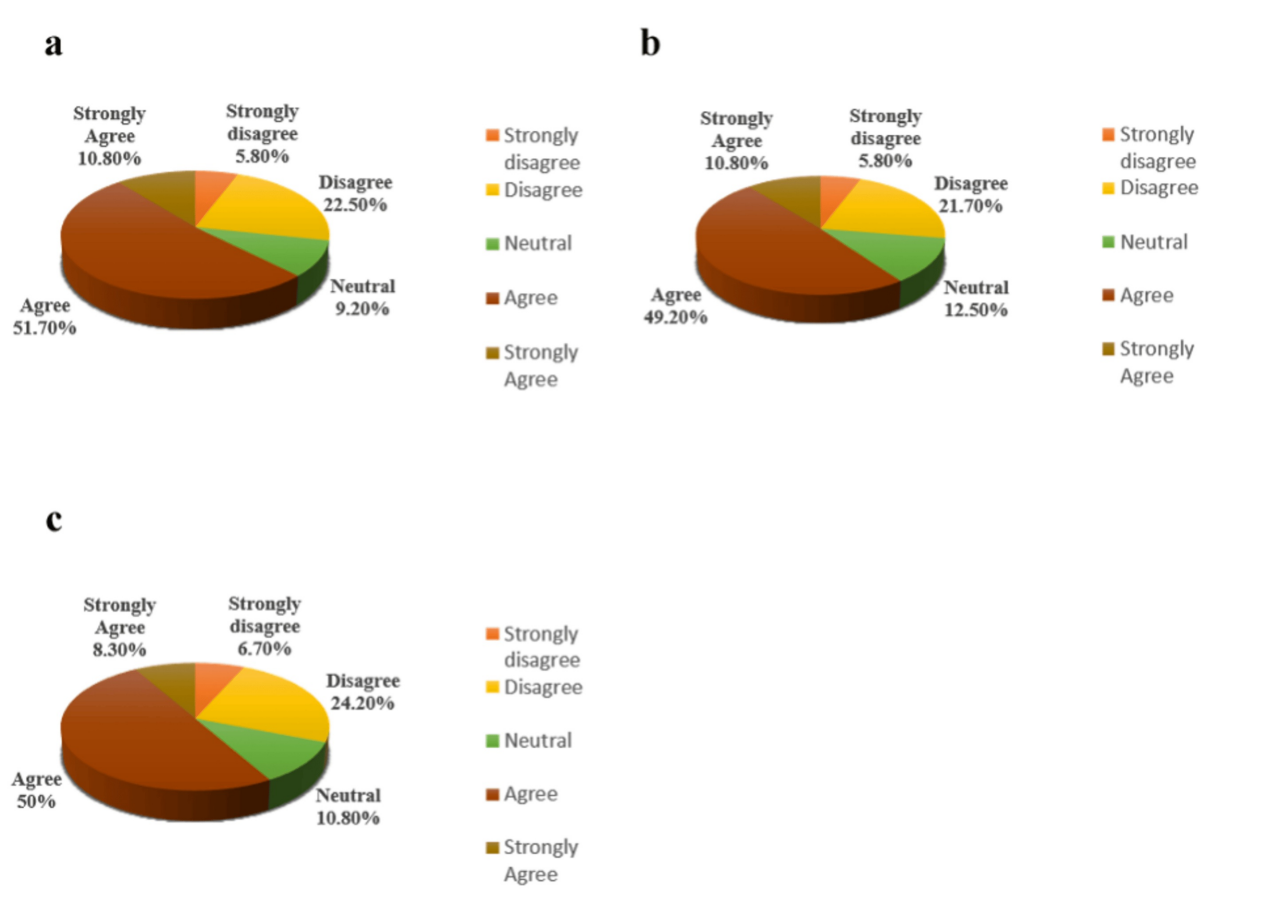
Students’ responses to the questionnaire’s representative parameters associated with online learning: part 3. (a) Online education would make it easy for slow learners and shy students to interact better. (b) A sufficient number of questions, or multiple-choice questions (MCQs), are asked by teachers to make online learning sessions interactive. (c) It simply takes more time to effectively accomplish tasks in an online environment than in a face-to-face class.

Regarding internet accessibility and the feasibility of e-learning, 61.6% (satisfied group) of students reported being able to easily access the internet for their online studies, and 28.4% (dissatisfied group) encountered issues related to internet access, while 10% of students had an indifferent viewpoint (Figure 1a). Notably, 61.7% of students preferred online learning at home (Figure 1b), while only 27.5% expressed greater comfort with offline lectures in class (Figure 1c). Furthermore, 62.5% of students (dissatisfied group) believed that the nature of learning differs between online and traditional lecture hall settings (Figure 1d). Regarding engagement, 61.7% of students indicated ease in asking questions during online classes (Figure 2a), with a corresponding belief among 61.7% of respondents that more questions can be asked in online classes compared to traditional settings (Figure 2b). Satisfaction with the organization and preparedness of classes was reported by 60.8% of respondents (Figure 2c), and 60% of students noted receiving quick responses from teachers when clearing queries online (Figure 2d). Additionally, 62.5% of participants believed that online learning facilitates better interaction for slow learners and shy students (Figure 3a). Participants also indicated a desire for interactive learning sessions, with 60% stating that teachers posed a sufficient number of questions or Multiple-Choice Questions (MCQs) during online sessions (Figure 3b). However, 58.3% of students felt that tasks took longer to complete successfully in an online environment compared to face-to-face classes (Figure 3c) (Table 1).

**Figure 4:**
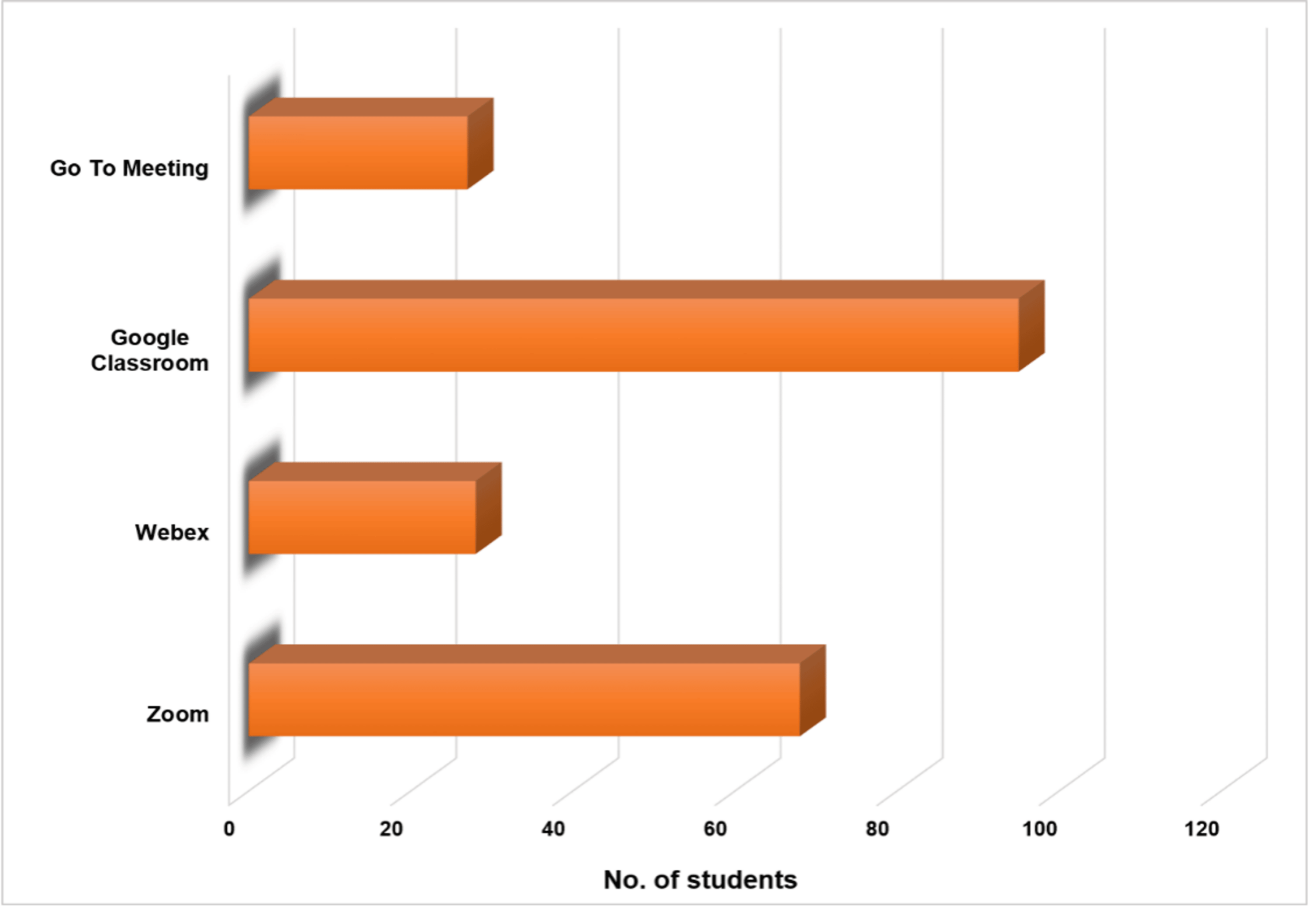
Preferred web conferencing platforms.

When asked about the preferred application for e-learning, Google Classroom was the preferred web conferencing platform, followed by Zoom (Figure 4).

**Figure 5:**
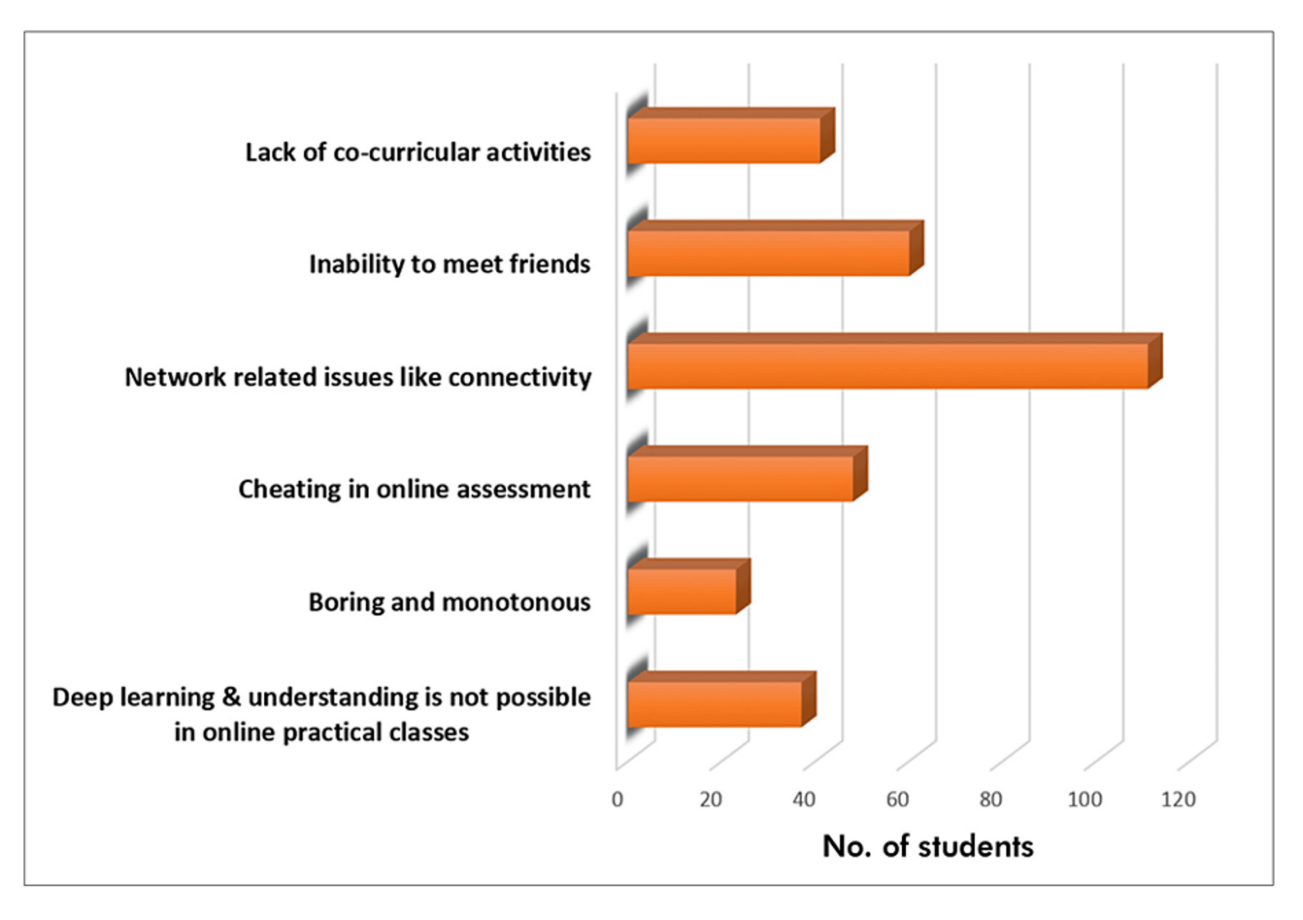
Problems faced by the students during e-learning.

The primary challenges encountered during e-learning were network-related issues, particularly internet connectivity, which 111 out of the 120 students stated. This was followed by the inability to meet friends, which was the issue faced by about half of the participants (Figure 5).

**Figure 6:**
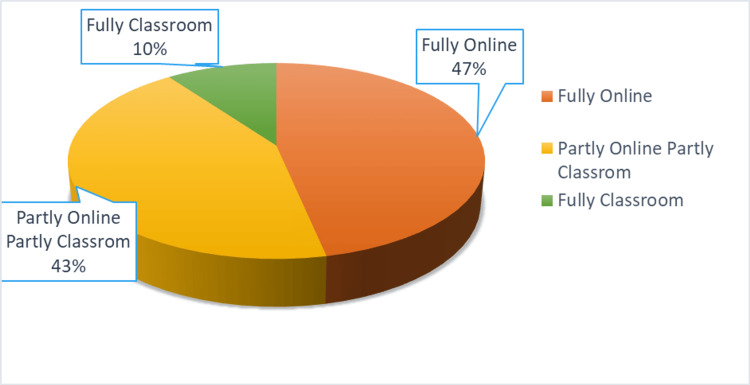
Student’s perception of the preferred mode of teaching in the future.

About 47% of the students showed a preference for online teaching in the future. While only 10% of participants favored offline teaching, 43% favored a mix of online and classroom teaching (Figure 6).

## Discussion

By replacing traditional teaching strategies with more creative, technologically driven, collaborative, and self-directed learning, COVID-19 has assisted in changing pedagogy. Our study, which involved second-year MBBS students of Silchar Medical College and Hospital, Assam, was done to assess the students' experience and perception of online learning during the COVID-19 pandemic. Our findings aligned with a similar study conducted at King Saud University, where 62% of students agreed that they could easily access the Internet for online learning [9]. Only 27.5% of students said they felt comfortable listening to an offline lecture in class, while 61.7% said they preferred online learning at home, which is almost identical to a survey done by Rayam et al. [10]. Singh et al. [8] found that 33% of students felt they had enough time to interact with their teachers during online classes. However, in the current study, most students said they could readily ask questions if they had trouble understanding an online lecture. Additionally, most students in our study believed that online learning made it easier for shy and slow learners to interact.

In January 2018, Vaona et al. [11] conducted a study on the e-learning of health professionals. They found that it may be more effective than traditional learning, particularly in medical education settings. A similar study in June 2018 by O’Doherty et al. [12], based on online learning in medical education, highlighted that online learning can provide a wider variety of content and more information more easily. Mamattah [13] also stated that, compared to traditional classroom learning, students expressed greater satisfaction with e-learning. Our findings align with the findings of these studies, and the students were more satisfied with online learning than with traditional classroom classes.

While most of the published literature talks about the advantages of e-learning, the challenges faced during online education also need to be analyzed in search of better teaching models for the future. In our study, network-related issues such as internet connectivity were the principal challenge faced by the students during online learning, followed by the inability to meet friends. A similar study also found that internet connectivity was the principal challenge faced by students during online learning [14]. In April 2019, Shrivastava et al. [15] published a study on challenges to e-learning in medical education. They reported that inadequate human resources are the biggest obstacle, while our study setting did not have this limitation. The major challenges faced in our study related to online teaching were the usage of newer technology, internet connectivity, inability to meet friends, and lack of co-curricular activities.

In a 2021 study, 81.1% of students felt online classes lacked the classroom atmosphere, and only 20% thought e-learning could replace traditional teaching [8]. However, our study reflects that the COVID-19 pandemic has caused a paradigm shift in learners' mindsets. Despite the problems faced, about 90% of students in our study preferred online or hybrid (a combination of online and classroom) modes of teaching, reflecting their acceptance of e-learning. Our findings align with a similar study on 161 students in a medical college in New Delhi, which revealed that most students do not prefer online or e-teaching alone, most likely due to a lack of interpersonal interaction. However, their preference for a hybrid mode of teaching was notable. Hybrid teaching holds the future of medical education as it is more student-friendly and efficient [3].

Exploring our findings with existing literature sheds light on the fact that the expectations and perceptions of students towards e-learning differ greatly based on the study setting. Research also points to the availability of resources and the affluence of the students' families that greatly influence the outcome of such studies from an Indian perspective [5]. A lack of affordable infrastructure, and, in certain cases, a lack of even the basic infrastructure needed for supporting e-learning, influences perceptions, mainly about the feasibility and accessibility of online education.

Our study has certain limitations, specifically the restricted sample size, the study setting being a single hospital, and the participants being only second-year MBBS students. The study also did not consider demographic factors influencing the accessibility of online education, such as the affluence of their family, availability of resources, etc. A single study setting also does not encompass the geographical or location factors that influence the accessibility and, thus, experience and perception of online education.

Future research may focus on comparative studies that can be undertaken with a larger niche. A study involving various study settings considering varied demographic factors would also shed light on the prevalence of challenges and help enlist improvements needed in our existing educational system to facilitate better learning infrastructure and adapt the best teaching mode. While this study focuses on the challenges and advantages of online learning from the student’s perspectives, future studies emphasizing the overall perspectives of students, teachers, and administrators would build a clearer picture. Further, developing an instrument to assess the effectiveness of online education will result in more standardized comparisons. Moreover, developing criteria for evaluating the instructional design of the online courses in MBBS educational institutions would greatly mediate the gap between the perceived appropriateness of online education and the actual educational outcomes.

## Conclusions

This study revealed the positive attitude of second-year MBBS students towards e-learning. Even though online medical education during the COVID-19 era had certain drawbacks, students adapted better to innovative teaching and learning techniques. The study highlighted the preference of the majority of participants for an online or hybrid mode of learning, suggesting it could serve as a potential guide for future planning of teaching models. Moreover, this study provided insights into the challenges of online health education and, hence, would help create a better study environment.

## Appendices

Questionnaire

*It is mandatory to answer all the questions*

* Indicates a required question

1. I have been well explained about the study and I am willing to participate in this study and give information to my best.

I am aware that my personal details will be kept confidential. I accept to participate in the study.

Mark only one oval.

Accept

Decline

2. Initials (Please write the first letter of your first name, first letter of your middle name, and first letter of your surname)

3. Age (in years) *

4. Sex *

Mark only one oval.

Male

Female

5. Education *

Mark only one oval.

1st Year MBBS

2nd Year MBBS

3rd Year MBBS

4th Year MBBS

6. I am able to easily access the internet as needed for my online studies. *

Mark only one oval.

Strongly Disagree

Disagree

Neutral/Equivocal

Agree

Strongly Agree

7. I am more comfortable with online learning at home. *

Mark only one oval.

Strongly Disagree

Disagree

Neutral/Equivocal

Agree

Strongly Agree

8. I am more comfortable with offline lectures in class. *

Mark only one oval.

Strongly Disagree

Disagree

Neutral/Equivocal

Agree

Strongly Agree

9. Learning is similar to online or conventional lectures in lecture hall. *

Mark only one oval.

Strongly Disagree

Disagree

Neutral/Equivocal

Agree

Strongly Agree

10. I can easily ask questions if there are problems in understanding an online lecture. *

Mark only one oval.

Strongly Disagree Disagree Neutral/Equivocal Agree

Strongly Agree

11. More questions can be asked by me during online learning. *

Mark only one oval.

Strongly Disagree

Disagree

Neutral/Equivocal

Agree

Strongly Agree

12. I am satisfied with the organisation and preparedness of the class. *

Mark only one oval.

Strongly Disagree

Disagree

Neutral/Equivocal

Agree

Strongly Agree

13. I get a quick response from the teacher for clearing my queries via online mode. *

Mark only one oval.

Strongly Disagree

Disagree

Neutral/Equivocal

Agree

Strongly Agree

14. Online education would make it easy for slow learners and shy students to interact better.

Mark only one oval.

Strongly Disagree

Disagree

Neutral/Equivocal

Agree

Strongly Agree

15. A sufficient number of questions or Multiple Choice Questions are asked by the teacher to make online learning sessions interactive.

Mark only one oval.

Strongly Disagree

Disagree

Neutral/Equivocal

Agree

Strongly Agree

16. It simply takes more time to effectively accomplish tasks in an online environment than in a face-to-face class.

Mark only one oval.

Strongly Disagree

Disagree

Neutral/Equivocal

Agree

Strongly Agree

17. What is your preferred web conferencing platform/platforms? *

Tick all that apply.

Zoom

Webex

Google Classroom

Go To Meeting

18. What problem/problems do you face during online teaching? *

Tick all that apply.

Deep learning & understanding is not possible in online practical classes

Boring and monotonous

Cheating in online assessment

Network-related issues like connectivity

Inability to meet friends

Lack of co-curricular activities

19. What is your perception about the preferred mode of teaching in the future? *

Mark only one oval.

Fully online

Partly online partly classroom

Fully classroom

This content is neither created nor endorsed by Google Forms.
